# A Plant-Derived Flavonoid, Isobavachin, Promotes Osteogenesis and Alleviates Glucocorticoid-Induced Osteoporosis via Modulation of the ESR1-PI3K/Akt Signaling Pathway

**DOI:** 10.3390/molecules31122158

**Published:** 2026-06-18

**Authors:** Jingran Cui, Xuting Song, Heran Liu, Zhenhai Cui, Mengmeng Sun, Min He, Meiying Jin

**Affiliations:** 1 School of Integrated Chinese and Western Medicine, Changchun University of Chinese Medicine, No. 1035, Boshuo Rd, Jingyue Economic Development District, Changchun 130117, China; 24211709046@ccucm.edu.cn (J.C.); 23211060204@ccucm.edu.cn (X.S.); 25211602022@ccucm.edu.cn (H.L.); cuizh@ccucm.edu.cn (Z.C.); 2 Northeast Asia Research Institute of Traditional Chinese Medicine, Changchun University of Chinese Medicine, Changchun 130117, China; sunmm@ccucm.edu.cn; 3 The Jilin Province School-Enterprise Cooperation Technology Innovation Laboratory of Herbal Efficacy Evaluation Based on Zebrafish Model Organisms, Changchun University of Chinese Medicine, Jingyue Economic Development District, Changchun 130117, China

**Keywords:** Isobavachin, osteoporosis, zebrafish, network pharmacology, PI3K inhibitor

## Abstract

Background: Glucocorticoid-induced osteoporosis (GIOP) is marked by impaired osteogenesis and reduced bone formation. Isobavachin (IBA), a flavonoid from Psoralea corylifolia, shows multiple potentials in anti-inflammatory and bone metabolism regulations, but its effects against GIOP remain unclear. This study investigated the osteoprotective effects and potential mechanism of IBA using zebrafish GIOP model. Methods: osteoprotective effects of IBA was assessed by fluorescence imaging in a prednisolone-induced zebrafish model, following with osteogenic gene expressions measured by RT-qPCR. Potential targets and pathways of IBA was filtered and predicted by network pharmacology, molecular docking, and molecular dynamics (MD) simulations, and finally validated with a pharmacological rescue experiment using a PI3K-specific inhibitor. Results: IBA improved bone mineralization and upregulated osteogenesis-related genes. Network pharmacology identified the PI3K-Akt pathway as a key pathway, with ESR1, GSK3B, MTOR, and CCND1 as core targets. PI3K inhibition attenuated the osteoprotective effects of IBA and suppressed downstream osteogenic gene expression. Conclusions: IBA alleviates GIOP by modulating the ESR1-associated PI3K-Akt signaling pathway and may serve as a multi-target therapeutic candidate for osteoporosis.

## 1. Introduction

Glucocorticoids are a class of steroid hormones widely used for their anti-inflammatory effects in conditions such as rheumatoid arthritis and systemic lupus erythematosus. However, long-term administration can cause several side effects, most notably GIOP, which is characterized by progressive bone loss and impaired bone-forming capacity. The pathogenesis of GIOP involves complicated mechanisms, among which glucocorticoid-induced suppression of osteoblast differentiation and osteogenic transcriptional activity is recognized as a major contributor to bone loss [1]. Although advances have been made in anti-osteoporotic therapies (e.g., bisphosphonates, RANKL inhibitors, selective estrogen receptor modulators, and parathyroid hormone analogs, etc.), current treatment strategies primarily focus on inhibiting bone resorption, while remaining relatively limited in restoring glucocorticoid-impaired osteoblast function and osteogenic capacity [2]. Consequently, developing therapeutic strategies capable of rescuing glucocorticoid-induced osteogenic dysfunction remains a critical challenge in this field. In this context, bioactive compounds derived from traditional medicinal plants have attracted increasing attention as potential therapeutic agents for bone metabolic disorders [3,4,5].

Among naturally derived compounds, flavonoids have shown considerable potential in regulating bone remodeling and osteoblast function [6]. These compounds may regulate bone metabolism through estrogen-like effects and the modulation of bone-related signaling pathways [7]. IBA, a major active compound derived from the traditional Chinese medicinal herb Psoralea corylifolia, has been reported to exhibit multiple regulatory effects on anti-inflammatory regulation and bone-related cellular processes. Recently, in vitro studies have demonstrated that IBA can inhibit RANKL-induced osteoclast differentiation and modulate osteoblast function [8]. However, systematic investigations into its in vivo effects and underlying mechanisms in osteoporosis—particularly in glucocorticoid-induced inhibition of osteogenesis—remain lacking.

Given the complexity of osteogenic regulation under glucocorticoid stress, integrative approaches may facilitate the identification of signaling pathways associated with the osteoprotective effects of IBA. Network pharmacology provides a powerful framework for integrating compound targets with disease-associated gene networks, enabling the identification of key regulatory nodes and construction of core signaling pathways [9]. Furthermore, molecular docking and MD simulations allow evaluation of ligand-receptor interactions at the structural level, providing additional support for mechanistic insights [10]. Therefore, integrating systems-level analysis with in vivo validation offers a comprehensive strategy to uncover the mechanisms of IBA.

Zebrafish are characterized by optical transparency, high fecundity, and suitability for high-throughput screening, making them an ideal model for in vivo studies. Importantly, key signaling pathways involved in bone development are highly conserved between zebrafish and mammals [11]. In osteogenesis research, zebrafish models enable comprehensive evaluation through multiple indicators, including bone mineralization, osteoblast activity, and osteogenesis-related gene expression. Notably, transgenic zebrafish expressing the osteoblast-specific transcription factor *sp7* allow for real-time visualization of osteoblast activity via fluorescent signals [12]. Additionally, prednisolone (Pred) exposure effectively recapitulates glucocorticoid-induced osteogenic suppression in zebrafish larvae, providing an in vivo platform for evaluating compounds with potential osteoprotective activity.

Based on the above background, this study aims to systematically evaluate the osteoprotective effects of IBA against glucocorticoid-induced osteogenic impairment, via both the phenotype and gene expressions in a prednisolone-induced transgenic zebrafish larva of the Tg(ola.*sp7*:nlsGFP) line, which specifically expresses green fluorescent protein in osteoblasts and offers a powerful tool for real-time visualization of bone formation and bone metabolism. In parallel, network pharmacology, molecular docking, and MD simulations were performed to identify candidate targets and signaling pathways potentially associated with the observed osteoprotective effects. Further inhibitor experiments were also conducted to confirm the regulatory effects of IBA on the identified core pathways and core targets. Our results demonstrate that IBA significantly improves bone mineralization and promotes the expression of osteogenesis-related genes, which effects are associated with modulation of the ESR1-mediated PI3K/Akt signaling pathway.

## 2. Results

### 2.1. Evaluation of the Biosafety of IBA and Its Osteoprotective Effect on the GIOP Zebrafish Model

**Figure 1:**
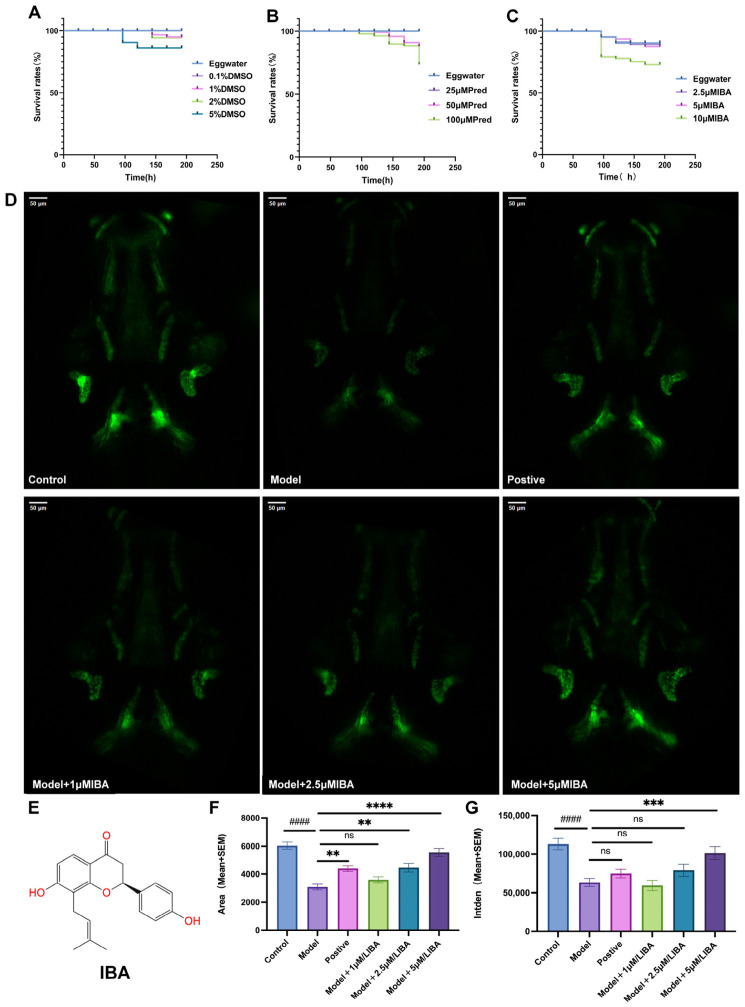
Biosafety and osteoprotective activity of IBA on the GIOP zebrafish model. (**A**) Biosafety assessment of DMSO; (**B**) biosafety assessment of Pred; (**C**) biosafety assessment of IBA; (**D**) representative fluorescence imaging results of zebrafish craniofacial skeletal regions under different treatment conditions; (**E**) chemical structure of IBA; (**F**) statistical analysis based on Fluorescence area; (**G**) statistical analysis based on fluorescence intensity. Compared with the control group, the significant difference is marked as: #### (*p* < 0.0001); compared with the model group, ** (*p* < 0.01), *** (*p* < 0.001), **** (*p* < 0.0001); ns: no statistically significant difference.

Priority to the activity evaluations, the toxicity of the solvent DMSO and the modeling chemical Pred was first systematically assessed in zebrafish larva, ensuring the reliability and biosafety of IBA efficacy evaluation. The results showed that although with dose-dependent decrease in survival rate, the DMSO solvent is still relative safe (with higher than 80% survival rate) within 196 hpf for zebrafish larvae when its concentration is lower than 5% (Figure 1A). Considering the later activity tests, 0.1% DMSO was selected as the final solvent concentration for subsequent experiments. Then, the biosafety of Pred and IBA was further evaluated separately, aiming to determine appropriate concentrations for GIOP modeling and subsequent intervention studies. Similarly, dose-dependent survival rate of Pred and IBA was also observed respectively. However, Pred with concentration below 100 μM (survival rate higher than 70%) and IBA with concentration below 10 μM (survival rate higher than 95%) were considered as relative safe for zebrafish larvae, over the observation period up to 196 h post-fertilization (hpf) (Figure 1B,C). In addition to the survival rate changes, no other significant morphological abnormalities, including developmental defects, body length differences, edema, or behavioral changes, were observed. Based on these safety assessments and dose–response pilot experiments, Pred with 25 μM was selected as the optimal concentration for model induction, and IBA with 1, 2.5, and 5 μM were selected as the final low, middle and high dosage respectively for the subsequent osteoprotective evaluations in zebrafish larvae.

Following the safety tests, a GIOP zebrafish model was established. Compared with the control group, zebrafish larvae treated with 25 μM Pred exhibited a significant reduction in both fluorescence area and fluorescence intensity in the skeletal regions (Figure 1D), indicating decreased bone mineralization and inhibited osteogenesis. These results confirm that 25 μM Pred significantly induced an osteoporosis-like phenotype in zebrafish under the present experimental conditions. IBA is a flavonoid (Figure 1E), which may play its osteoprotective effects through estrogen-like effects. Using such a model, the osteoprotective effects of IBA were assessed. Fluorescence imaging showed that IBA markedly alleviated Pred-induced bone loss. Compared with the model group, IBA treatment significantly increased both the fluorescence area and intensity in the craniofacial skeleton. Quantitative analysis further demonstrated a dose-dependent improvement in bone mineralization. Intergroup comparisons revealed that the 5 μM IBA group exhibited a significantly larger fluorescence area than both the 1 μM and 2.5 μM groups (*p* < 0.05), while fluorescence intensity was significantly higher than that of the 1 μM group (*p* < 0.01). The 5 μM IBA group showing the strongest effect, comparable to that of the positive control, etidronate disodium (10 μg/mL) (Figure 1F,G). Collectively, these results indicate that IBA exerts a significant and dose-dependent protective effect against glucocorticoid-induced bone loss in zebrafish.

### 2.2. The Effect of IBA on the Transcription Level of Zebrafish Bone-Related Genes

**Figure 2:**
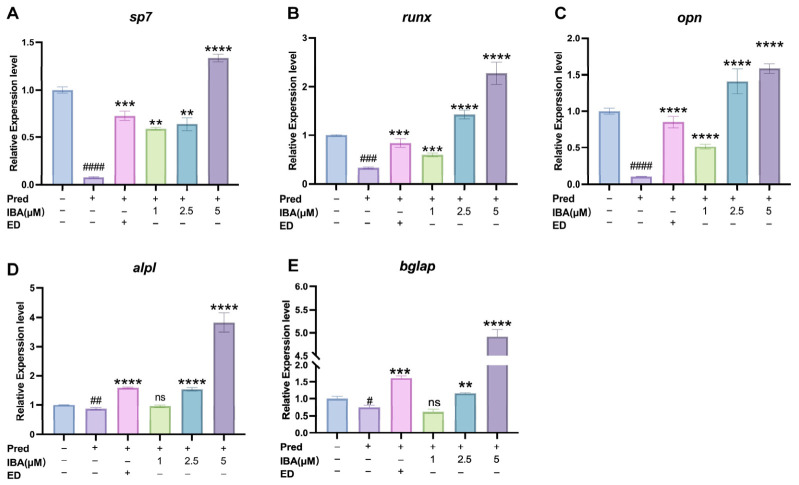
IBA regulates the expression of osteoporosis-related genes. Detect bone-related factors: (**A**) *sp7*; (**B**) *runx*; (**C**) *opn*; (**D**) *alpl*; (**E**) *bglap* by RT-qPCR. The bar chart shows the average ± standard error (SEM) calculated by three independent experiments. Compared with the control group, the significant difference is marked as: # (*p* < 0.05), ## (*p* < 0.01), ### (*p* < 0.001), #### (*p* < 0.0001); compared with the model group, ** (*p* < 0.01), *** (*p* < 0.001), **** (*p* < 0.0001); ns indicates that there is no statistically significant difference.

To further elucidate the osteogenic effect of IBA at the molecular level, quantitative real-time PCR (RT-qPCR) was performed to assess the mRNA expression levels of key osteogenic marker genes, including *alpl*, *spp1 (opn)*, *sp7*, *runx2*, and *bglap*, in zebrafish larvae. The results showed that, compared with the model group, IBA treatment significantly upregulated the mRNA expression levels of all examined osteogenesis-related genes (*p* < 0.05). Quantitative analysis further demonstrated a clear dose-dependent trend, in which the expression levels of *sp7*, *alpl*, *runx2*, *spp1 (opn)*, and *bglap* progressively increased with increasing concentrations of IBA (Figure 2A–E). Collectively, these findings indicate that IBA promotes osteogenesis in zebrafish by activating the transcription of key bone formation-related genes.

### 2.3. Target Screening and Prediction of IBA Anti-Osteoporosis Based on Network Pharmacology

#### 2.3.1. Interaction (PPI) Network Construction and KEGG Pathway Enrichment Analysis of Potential Targets

**Figure 3:**
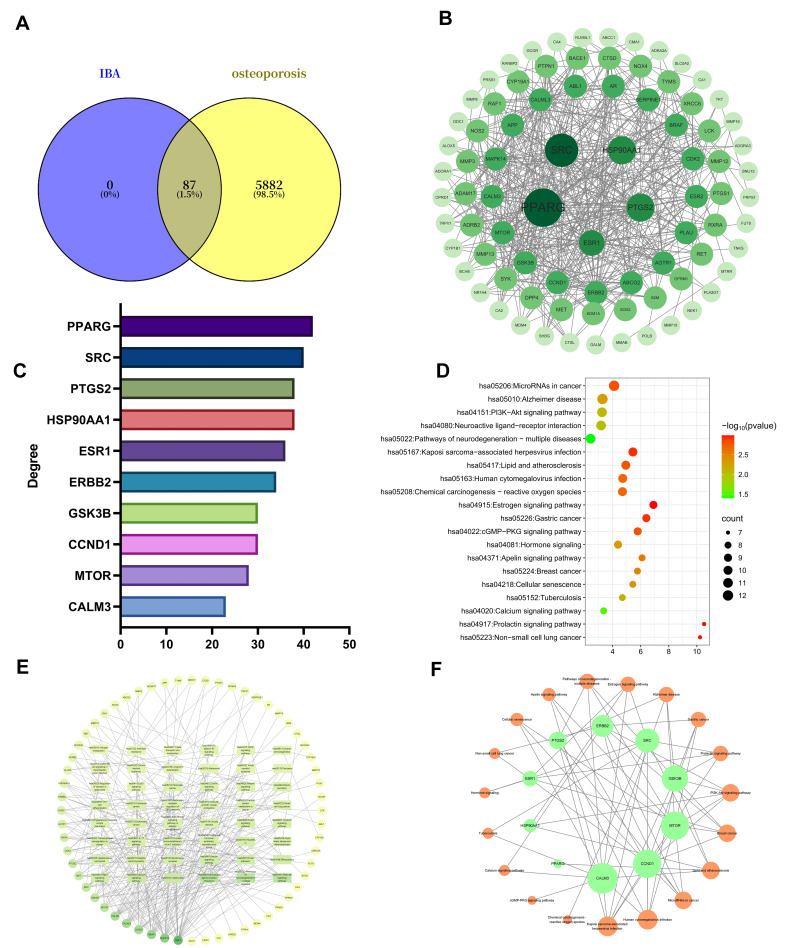
Analysis of the mechanism of the potential effect of IBA on osteoporosis. (**A**) The results of the Venn chart analysis of the common target of IBA and osteoporosis; the purple area on the left represents the target information of IBA, the yellow area on the right represents the target information of osteoporosis, and the overlapping part represents the intersection of the two; (**B**) PPI (protein interaction) network diagram of IBA intersection target, the size of the node represents the degree value; the higher the degree value, the larger the node and the darker the color, suggesting that its core position in the network is stronger; (**C**) top 10 key target genes in the frequency value; (**D**) key signal pathway enrichment analysis bubble diagram (sorted by enrichment value); (**E**) target-pathway quantity frequency relationship diagram in KEGG pathway enrichment analysis, The color intensity of the nodes represents the relative frequency of target occurrence, with darker colors indicating higher frequencies.; (**F**) core target-KEGG core channel quantity frequency relationship diagram, Green nodes represent core targets and orange nodes represent enriched KEGG pathways.

By integrating the TCMSP, Swiss Target Prediction, and PharmMapper databases, a total of 87 potential targets of IBA were predicted. Concurrently, 5969 osteoporosis-related targets were collected from the OMIM and GeneCards databases using the keyword “osteoporosis.” Intersection analysis between IBA targets and osteoporosis-related targets identified 87 overlapping genes (Figure 3A), suggesting that these targets may mediate the regulatory effects of IBA on osteoporosis-related biological processes. To further investigate the interactions among these intersection targets, the 87 common targets were imported into the STRING database (species limited to Homo sapiens) to construct a protein–protein interaction (PPI) network (Figure 3B). The results revealed extensive interactions among these targets, forming a complex protein interaction network. Network topology analysis, using parameters such as degree, identified the top 10 key targets with the highest connectivity, including PPARG, SRC, PTGS2, HSP90AA1, ESR1, ERBB2, GSK3B, CCND1, mTOR, and CALM3 (Figure 3C). Subsequent KEGG pathway enrichment analysis showed significant enrichment of the PI3K-Akt signaling pathway, hormone-related signaling pathways, and other pathways (Figure 3D). Target–pathway correlation analysis further indicated that the PI3K-Akt signaling pathway exhibited the highest degree of association with the intersection targets, suggesting that it may serve as the core regulatory axis mediating the anti-osteoporotic effects of IBA (Figure 3E).

Combined with the results of PPI network topology analysis, the core targets were predominantly enriched in signaling pathways such as the PI3K-Akt and Estrogen signaling pathways, with key nodes such as mTOR and CCND1 participating in the PI3K-Akt-related network (Figure 3F, Table 1). These results indicate that the PI3K-Akt signaling pathway is likely the key pathway underlying IBA’s anti-osteoporotic activity, as supported by both pathway enrichment and network topology analyses. Accordingly, the PI3K-Akt signaling pathway was selected as the focus for subsequent mechanistic studies. Within this framework, ESR1, PIK3CA, AKT1, GSK3B, mTOR, and CCND1 were identified as candidate core targets by integrating PPI network degree values and relevant literature on bone metabolism regulation. Functionally, these targets occupy different regulatory positions within the PI3K-Akt signaling pathway: ESR1 acts as an upstream hormone receptor, PIK3CA and AKT1 form the signal transduction core, while GSK3B, mTOR, and CCND1 function as downstream effectors. Collectively, they regulate cell proliferation and osteoblast differentiation, constituting a key molecular axis controlling bone metabolism.

In summary, based on a triple-screening strategy of pathway enrichment analysis- network topology-disease correlation, these six targets were identified as the key candidates for subsequent molecular docking and mechanistic studies.

#### 2.3.2. GO Functional Enrichment Analysis

**Figure 4:**
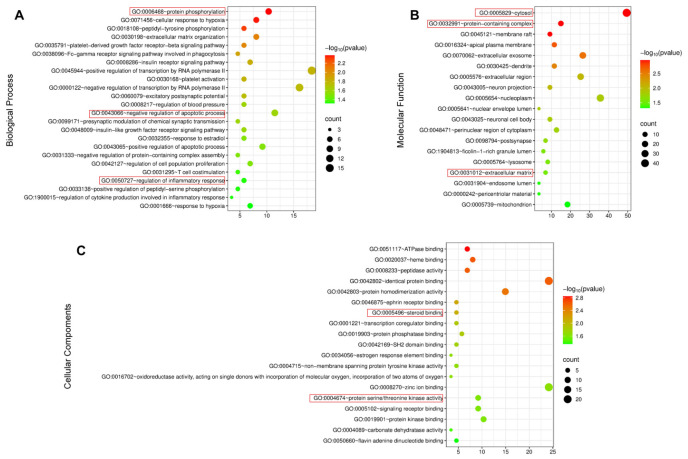
GO function enrichment analysis of IBA and osteoporosis intersection targets. (**A**) Biological process (BP); (**B**) cell components (CC); (**C**) molecular function (MF). The bubble size indicates the number of enriched genes (Count), the color gradient indicates the enrichment significance (−log10*p*value), and the horizontal axis represents GeneRatio. Terms highlighted by red boxes were considered particularly relevant to the subsequent mechanistic analysis.

To clarify the biological functions underlying the anti-osteoporotic effects of IBA, a Gene Ontology (GO) functional enrichment analysis was performed on the 87 overlapping targets. At the Biological Process (BP) level, these targets were significantly enriched in processes such as the regulation of protein phosphorylation, cell signal transduction, extracellular matrix organization, regulation of inflammatory response, and the modulation of cell proliferation and apoptosis (Figure 4A). Regarding Molecular Function (MF), the results indicated that the targets are primarily involved in protein kinase activity, protein kinase binding, SH2 domain binding, steroid binding, and the regulation of transcription (Figure 4B). In terms of Cellular Component (CC), the target proteins were predominantly localized in the cytoplasm, cell membrane domains, protein complexes, and the extracellular matrix (Figure 4C).

### 2.4. Molecular Docking and Visual Analysis of IBA and Core Targets

In order to verify the affinity between IBA and the screened core targets at the molecular level, this study used molecular docking technology to simulate the interaction modes between IBA and the above-mentioned six key proteins. Molecular docking results showed that IBA and the six target proteins exhibited good binding ability, and the binding energy of all complexes was lower than −7.0 kcal/mol, suggesting that IBA and these proteins have a relatively stable binding affinity. Among them, the binding energy of IBA and mTOR was the lowest (−10.4 kcal/mol) (Table 2), showing the highest binding affinity. In addition, IBA also showed excellent complementarity with the signal transduction core protein AKT1 and its downstream effector molecule GSK3B.

**Figure 5:**
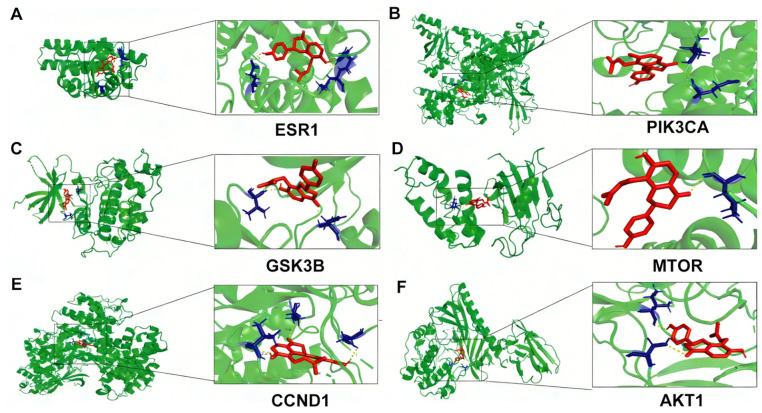
Molecular docking analysis of IBA-protein target complex. (**A**–**F**) are the composite structure diagrams of IBA and ESR1, PIK3CA, GSK3B, mTOR, CCND1 and AKT1 proteins respectively. The protein structures are shown in green, IBA is shown in red, and the interacting amino acid residues are shown in blue.

Further visualization analysis of the binding mode showed that IBA can be embedded into the active pocket of the target protein and form a stable complex through a variety of non-covalent interactions, such as hydrogen bonds, hydrophobic interactions, and π–π stacking (Figure 5). The above results show that IBA has a good binding ability with multiple key regulatory proteins, providing structural support for its regulation of bone metabolism-related signaling networks.

### 2.5. MD Simulation Analysis of the Binding Stability of IBA and Core Target Complex

In order to further evaluate the dynamic stability of IBA-target protein complexes, this study conducted a 100 ns MD simulation of the six complex systems mentioned above.

**Figure 6:**
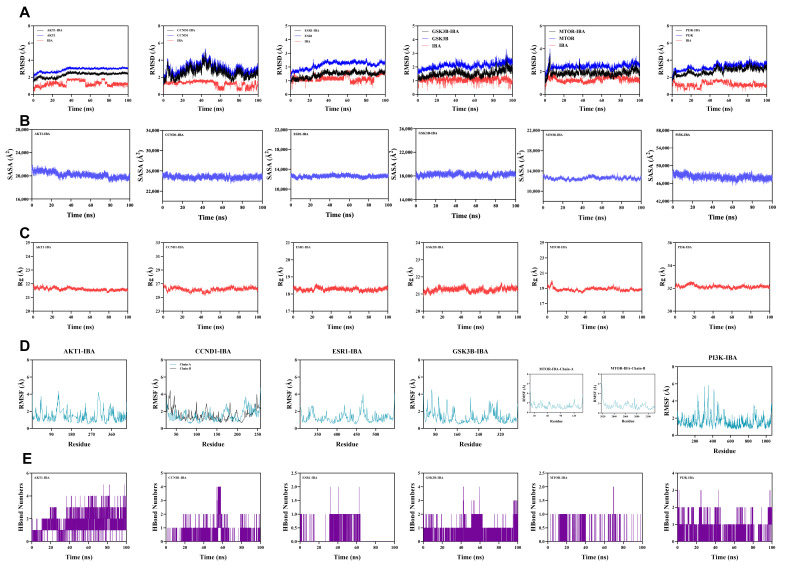
MD simulation of protein–ligand complex. (**A**) RMSD change curve of protein–ligand complex. (**B**) Rg change curve of protein–ligand complex. (**C**) SASA change curve of protein–ligand complex. (**D**) RMSF distribution of protein–ligand complex residues. (**E**) Hydrogen bonds.

The root mean square displacement (RMSD) results showed that most complex systems gradually reached a stable state during the simulation process (Figure 6A). Among them, the AKT1-IBA complex tended to stabilize after about 35 ns and fluctuated around 2.5 Å; the CCND1-IBA complex stabilized after about 90 ns and remained around 5 Å; the ESR1-IBA complex stabilized after about 80 ns with an RMSD of about 1.8 Å; the GSK3B-IBA complex stabilized at about 2.0 Å after about 85 ns; the mTOR-IBA complex quickly reached a stable state after about 5 ns and remained at about 2.3 Å; and the PIK3CA-IBA complex stabilized at about 3.5 Å. The radius of gyration (Rg) analysis showed that each complex system exhibited only slight fluctuations during the simulation (Figure 6B), indicating that the overall structure of the proteins remained relatively stable. The solvent accessible surface area (SASA) analysis showed that the overall change in each complex system was relatively small during the simulation (Figure 6C), suggesting that ligand binding did not cause obvious changes in protein surface exposure. The root mean square fluctuation (RMSF) curve of each complex showed that, except for some loop regions, the fluctuations of most amino acid residues were relatively small, indicating that the protein skeleton maintained good structural rigidity after binding to IBA (Figure 6D). In MD simulations, hydrogen bonding plays a critical role in ligand–protein association. Figure 6E illustrates the fluctuation ranges and predominant hydrogen-bond counts between IBA compounds and their respective target proteins. For the AKT1-IBA complex, the number of hydrogen bonds varies from 0 to 5, with approximately 3 bonds observed most frequently. In contrast, CCND1-IBA and GSK3B-IBA exhibit a range of 0 to 4 hydrogen bonds, whereas ESR1-IBA, MTOR-IBA, and PI3K-IBA show narrower ranges of 0 to 2 (or 3). In these latter five complexes, only about 1 hydrogen bond is maintained in the majority of conformations. (Figure 6E). These findings indicate that IBA forms a relatively stable and numerous hydrogen-bond network with AKT1, yet engages in markedly weaker hydrogen-bonding interactions with the other targets, underscoring distinct binding modes across different receptors. Specifically, the RMSF values of most residues in the AKT1-IBA, CCND1-IBA, and GSK3B-IBA complexes were less than 4 Å; the residue fluctuations of the ESR1-IBA and mTOR-IBA complexes were mainly lower than 3 Å, while those of the PIK3CA-IBA complex were mainly below 5.8 Å, indicating low overall residue flexibility.

In summary, the MD simulation results show that IBA and the above-mentioned target protein complexes maintain a relatively stable binding state during the simulation. However, computational simulation results alone are not sufficient to fully clarify the biological mechanism of action; the regulatory role of IBA in key signaling pathways still needs to be further verified through in vivo functional experiments.

### 2.6. IBA Based on PI3K Inhibitor Promotes Bone Action and Verification of Its Signal Transduction Mechanism

Based on the aforementioned integrated network pharmacology, molecular docking, and MD simulations, the “ESR1-PI3K/AKT-osteogenic differentiation” axis were filtered and considered as a potential pathway underlying the regulatory effects of IBA on bone metabolism. In this axis, ESR1 mediates hormone signal input as an upstream receptor, while the PI3K/AKT pathway acts as a key signal transduction core, affecting the bone formation process by regulating the expression of downstream osteogenesis-related genes (such as *ccnd1*, *runx2*, and *alpl*). To systematically verify this signaling axis during IBA-promoted bone mineralization, this study conducted verification at both functional and molecular levels. First, the PI3K/AKT pathway was functionally blocked using the PI3K-specific inhibitor LY294002 to evaluate its role in IBA-induced effects. Subsequently, RT-qPCR was used to detect expression changes in key molecules to clarify the molecular regulatory pathway.

**Figure 7:**
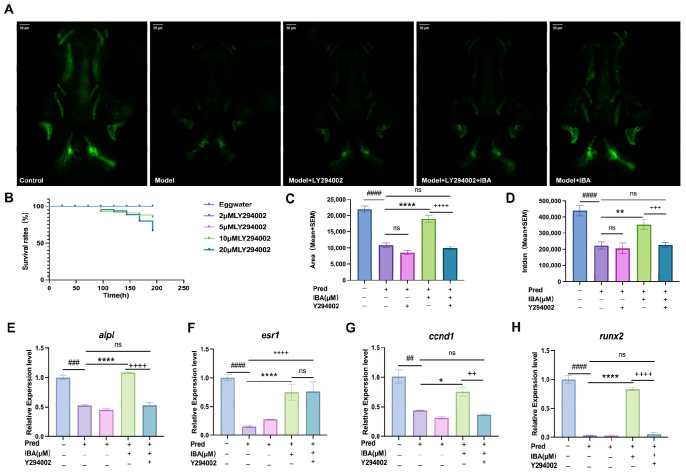
PI3K/AKT signal pathway function blocking and molecular mechanism verification. (**A**) Tg(*sp7*:EGFP) transgenic zebrafish bone mineralization fluorescence representative image; (**B**) security assessment of LY294002; (**C**) quantitative analysis of zebrafish bone mineralization fluorescence area of each experimental group; (**D**) quantitative analysis of zebrafish bone mineralization fluorescence intensity of each experimental group; (**E**–**H**) RT-qPCR detection *esr1*, *ccnd1*, *runx2* and *alpl*. The bar chart data is expressed as the average ± standard error (SEM) of the results of three independent experiments. Compared with the control group, ## (*p* < 0.01), ### (*p* < 0.001), #### (*p* < 0.0001); compared with the model group, * (*p* < 0.05), ** (*p* < 0.01), **** (*p* < 0.0001); compared with the treatment group, ++ (*p* < 0.01), +++ (*p* < 0.001), ++++ (*p* < 0.0001); ns indicates that the difference is not statistically significant.

Priority to the functional blocking experiments, the toxicity of LY294002 was evaluated. As shown in Figure 7B, at a concentration of 10 μM up to 196 hpf, the survival rate of zebrafish larvae was higher than 90%; however, when the concentration increased to 20 μM and above, the survival rate decreased significantly at 196 hpf. Therefore, 10 μM was determined as its working concentration for follow-up experiments. Under these concentrations, it was found that the bone fluorescence area and intensity of the zebrafish skeletal regions were significantly reduced in the IBA + inhibitor group (Figure 7A,C,D), compared with the IBA group. This result suggests that suppressing the PI3K signaling pathway via the LY294002, the promoting effect of IBA on bone mineralization was significantly attenuated in zebrafish. In another word, the PI3K signaling pathway plays a key mediating role in IBA-promoted bone mineralization and inhibited osteogenesis.

Further RT-qPCR results also supported our findings. Compared with the model group, IBA treatment significantly upregulated the mRNA expression levels of *esr1*, *ccnd1*, *runx2*, and *alpl* (*p* < 0.05). After IBA intervention combined with the LY294002, the expression levels of the down-stream genes (*ccnd1*, *runx2*, and *alpl*) were inhibited significantly, while there was no significant change in the up-stream gene expression (*esr1*) (Figure 7E–H). These results indicate that the PI3K/AKT signaling pathway mainly mediates the regulatory effect of IBA on downstream osteogenesis-related genes. This suggests that IBA may promote osteogenic differentiation by activating ESR1, which further regulates the PI3K/AKT pathway and downstream gene expression, ultimately exerting its bone-protective effects.

## 3. Discussion

This study systematically evaluated the osteoprotective effect of IBA, a bioactive flavonoid derived from natural products, in a GIOP model and further elucidated its multi-target regulatory mechanisms. We found that IBA significantly reversed Pred-induced mineralization damage in zebrafish and improved the osteoporosis phenotype by enhancing osteogenic activity. Network pharmacology analysis revealed that the PI3K-Akt signaling pathway serves as the core hub for IBA’s efficacy. Combined with molecular docking, MD simulations, and PI3K inhibitor intervention, this study further supported the involvement of the ESR1/PI3K-Akt signaling cascade in the osteoprotective effect of IBA. These findings underscore the potential of IBA as a multi-target natural therapeutic agent for osteoporosis.

### 3.1. IBA Promotes Osteogenic Differentiation by Activating Bone Formation-Related Genes

Osteogenesis is a complex biological cascade driven by specific transcription factors. The *sp7* is essential for the transition of pre-osteoblasts into mature osteoblasts [13], while *runx2*, the “master switch” of bone development, determines the lineage commitment of mesenchymal stem cells (MSCs) toward osteoblasts [14]. Glucocorticoids typically impair bone formation by inhibiting these two key transcription factors [15]. In this study, a Pred-induced osteoporosis model was established using Tg(ola.*sp7*:nlsGFP) zebrafish larvae. We observed that IBA significantly increased the fluorescence area and signal intensity in the cranium and axial bones in a dose-dependent manner. This phenotypic improvement demonstrates IBA’s ability to reverse bone mineralization damage. Furthermore, IBA significantly upregulated the expression of *runx2* and *sp7*. These results suggest that IBA successfully antagonizes the inhibitory effect of Pred on these factors, ensuring the differentiation potential of osteoblasts at the source.

Beyond differentiation, bone mineralization involves two critical processes: inorganic phosphate (Pi) release and mineral salt deposition [16]. In these processes, *alpl* and *opn* (*spp1*) play central roles: *alpl* hydrolyzes pyrophosphate (PPi) to provide Pi, while *opn* mediates the nucleation and adhesion of bone crystals via its phosphorylation sites and RGD sequences [17,18]. Our study found that IBA promoted the expression of *alpl* and *spp1*/*opn*. This synchronous upregulation supports the observed enhancement in zebrafish bone fluorescence, revealing IBA’s capacity to drive early matrix synthesis. Finally, the increased expression of the mature marker *bglap* confirms that IBA drives osteoblasts toward full physiological maturity.

In summary, the reparative effect of IBA is not limited to a single stage but is achieved through a coordinated “differentiation (*runx2*/*sp7*)–matrix synthesis (*alpl*/*spp1*)–final mineralization (*bglap*)” axis. Compared to previous reports focusing on IBA’s inhibition of osteoclast activity [8], this study highlights its role in promoting osteoblast differentiation, suggesting IBA is a dual-functional regulator of bone metabolism.

### 3.2. Network Pharmacology and Molecular Interaction Analyses Suggest the Involvement of the ESR1-PI3K/Akt Axis in the Osteogenic Effects of IBA

Utilizing systems biology through network pharmacology and molecular docking helps identify potential targets and pathways associated with the effects of IBA. Our analysis identified that IBA-related targets in GIOP are significantly enriched in the PI3K-Akt signaling pathway. Notably, core nodes such as ESR1, GSK3B, mTOR, and CCND1 form a logical chain from “receptor recognition” to “signal transduction” and “functional expression.” Molecular docking and MD simulations confirmed that IBA possesses strong binding affinity and dynamic stability with these targets. This structural consistency provides a solid basis for the hypothesis that IBA acts as a ligand-like molecule to activate this signaling axis.

Existing studies confirm that ESR1 is a primary upstream receptor regulating osteoblast function [19]. Upon activation, it can recruit and activate PI3K in the cytoplasm via non-genomic effects [20], triggering the AKT phosphorylation cascade [21]. As a central kinase, AKT regulates multiple downstream effectors: it inactivates GSK3B via phosphorylation, thereby preventing *β-catenin* degradation and activating Wnt/β-catenin signaling [22]; simultaneously, it activates mTOR to enhance protein translation and matrix synthesis [23]. Furthermore, CCND1, a key regulator of the cell cycle, promotes the transition of osteoprogenitors from G1 to S phase, maintaining the osteoblast pool [24].

**Figure 8:**
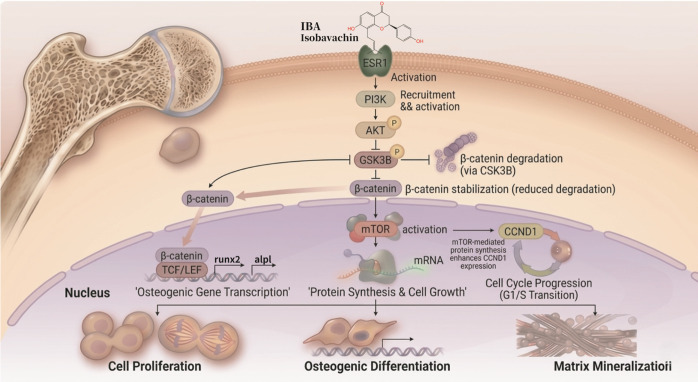
IBA-dependent osteogenesis mechanism of ESR1-PI3K/Akt axis. IBA activates the ESR1-PI3K/Akt signaling axis, leading to GSK3B inhibition, β-catenin stabilization, and mTOR activation. This cascade upregulates CCND1-driven cell cycle progression, osteogenic gene transcription (e.g., Runx2), and ultimately enhances osteoblast proliferation, differentiation, and matrix mineralization.

In conclusion, by integrating bioinformatics and functional validation, this study established a bone-protective signaling model involving ESR1, PI3K/AKT, and downstream effectors such as CCND1 and GSK3B. This coherent pathway explains how IBA reshapes bone homeostasis under glucocorticoid stress and provides a mechanistic entry point for its clinical potential. Rather than acting on a single target, IBA may exert multi-level osteogenic regulatory effects through an ESR1/PI3K/Akt-associated signaling pathway (Figure 8).

### 3.3. PI3K Inhibitor Validation Supports the Involvement of the PI3K/Akt Pathway in the Osteogenic Effects of IBA

This study further verified the reliability of the network pharmacology predictions through PI3K-specific inhibitor rescue experiments, providing functional evidence for the involvement of the PI3K-Akt pathway in IBA’s efficacy. As a central signaling hub involved in osteogenic regulation, PI3K integrates multiple upstream signals and transduces them to downstream effectors associated with bone formation. By pharmacologically blocking this key signaling node, we sought to determine whether the osteoprotective effects of IBA were dependent on PI3K/Akt pathway activity.

Our results support that IBA’s ability to improve the bone phenotype is highly dependent on the activation of the PI3K-Akt signaling pathway. When the pathway was blocked, IBA significantly lost its bone-protective activity, as evidenced by the down regulation of key downstream genes such as *runx2*, *alpl*, and *ccnd1*, and the regulation of ESR1 by IBA was not affected by the inhibitor. This suggests that the PI3K-Akt pathway is an important signaling route involved in the osteoprotective effects of IBA, with PI3K/Akt functions downstream of ESR1 and upstream of osteogenic effectors. However, additional studies are required to determine whether ESR1 directly mediates the biological activity of IBA.

The regulatory effect of IBA on the ESR1/PI3K-Akt-associated signaling axis is supported by other similar flavonoids research. For instance, Daidzein has been found to trigger non-genomic effects via the ESR1-mediated PI3K-Akt pathway [25]. Additionally, Lycopene significantly induces osteogenic differentiation and improves osteoporosis phenotypes through this same axis [26]. This consistency across different compounds and models further supports the potential involvement of ESR1-associated PI3K/Akt signaling in osteogenic regulation.

### 3.4. The Advantages and Prospects of IBA as a Natural Multi-Target Bone Protector

As a bioactive flavonoid derived from natural sources, IBA exhibits distinct pharmacological advantages characterized by multi-target regulation and pathway-level coordination. The ESR1-PI3K/Akt signaling axis not only transmits instructions for bone formation but may also act as a hub for regulating microenvironmental homeostasis during the bone repair process. In terms of inflammatory regulation, activated ESR1 can influence cytoplasmic signaling through non-genomic effects, and its downstream PI3K/Akt pathway has been proven to negatively regulate inflammatory factors in various models [27]. Additionally, IBA has been reported to suppress NF-κB signaling [28]. This suggests that IBA may promote osteogenesis while simultaneously improving the chronic inflammatory environment induced by glucocorticoids. This potential dual effect based on the ESR1-PI3K/Akt axis may be a significant advantage of IBA over single-target synthetic drugs. Furthermore, IBA has shown potential in antioxidant and mitochondrial protection; it was reported to inhibit mitochondrial biosynthesis in bone loss models caused by periodontitis and activate Nrf2 to combat liver damage [8]. These biological processes often involve the PI3K/Akt-mediated phosphorylation of downstream effectors, suggesting that IBA possesses broader systemic cytoprotective potential.

It is worth noting that dysregulation of the ESR1-PI3K/Akt pathway has also been reported in several metabolic disorders beyond osteoporosis, including conditions associated with impaired glucose and lipid metabolism [29,30]. These observations suggest that this signaling pathway may have broader physiological relevance. However, the present study focused exclusively on glucocorticoid-induced osteoporosis, and the potential effects of IBA in other disease settings require further investigation [31].

From a network perspective, the ESR1-PI3K/Akt pathway exhibits a consistent “pro-survival and pro-metabolic” function across bone, liver, and skeletal muscle tissues. Its insufficiency is a common pathological feature of osteoporosis, type 2 diabetes, NAFLD, and myasthenia. Therefore, beyond the osteogenic effect on GIOP found in this study, IBA may have the potential to treat the aforementioned metabolic diseases through the principle of “treating different diseases with the same method”. The present study was performed in a larval zebrafish model, which provided an efficient platform for screening and discovering the potential anti-osteoporotic activity of IBA and exploring its potential mechanism of action. These findings support the early-stage development of IBA as a natural product candidate; while further validation in mammalian GIOP models is still required before its therapeutic potential can be fully established. In addition, although LY294002 is widely used as a pharmacological inhibitor of PI3K/Akt signaling, future studies employing more selective genetic approaches will be valuable for further validating the involvement of this pathway in the osteoprotective effects of IBA. In summary, IBA does not act through a single molecular target but achieves coordinated regulation of bone formation, inflammation, and oxidative stress via the ESR1-PI3K/Akt signaling axis. This “multi-target, pathway-oriented” mode of action highlights the unique advantages of natural flavonoids and provides a mechanistic basis for the development of novel phytochemical-based therapeutic strategies in osteoporosis and related metabolic disorders.

## 4. Materials and Methods

### 4.1. Chemicals and Reagents

Isobavachin (IBA), dimethyl sulfoxide (DMSO), tricaine, and etidronate disodium were purchased from Shanghai Yuanye Biotechnology Co., Ltd. (Shanghai, China). Prednisolone (Pred) was obtained from Shanghai Linen Technology Development Co., Ltd. (Shanghai, China). TRIzol Reagent was purchased from Ambion, Inc. (Austin, TX, USA), and the PI3K-specific inhibitor LY294002 was purchased from Shanghai Aladdin Biochemical Technology Co., Ltd. (Shanghai, China). All other reagents were purchased from Tianjin Dingfu Chemical Co., Ltd. (Tianjin, China). The purity of all reagents was ≥98%.

### 4.2. Experimental Animals

Wild-type AB strain zebrafish and the osteoblast-specific fluorescent transgenic line Tg(ola.*sp7*:nlsGFP) were used in this study. All zebrafish were maintained under standard husbandry conditions in accordance with the guidelines of the Zebrafish Model Organism Database (ZFIN). The experimental protocol was approved by the Experimental Animal Welfare and Ethics Committee of Changchun University of Traditional Chinese Medicine (Approval No. 20255345).

Zebrafish were maintained under a 14 h light/10 h dark cycle at 28 °C. Healthy adult zebrafish (6–12 months old) were selected for natural mating to obtain fertilized embryos. The collected embryos were cultured in embryo medium containing 0.2 g/L instant sea salt and 0.0025% methylene blue for subsequent experiments.

### 4.3. Safety Evaluation of Solvent DMSO, Modeling Chemical (Pred), Natural Product IBA, and PI3K-Specific Inhibitor LY294002

Three days post-fertilization (dpf) zebrafish larvae were randomly divided into groups (*n* = 30 per group) and exposed to DMSO at different concentrations (0%, 0.1%, 1%, 2%, and 5%; 0% served as the control). The medium was refreshed every 24 h and Dead and unfertilized embryos were removed to keep the good condition for later experimental evaluations. After determining the safe concentration of DMSO, Pred, IBA, and LY294002 were dissolved using the safe dosage of the DMSO. Larvae were exposed to IBA (2.5, 5, 10 μM) and LY294002 (2, 5, 10, and 20 μM), respectively. Survival rate and morphological changes were assessed as described above to determine the safe concentrations for subsequent experiments. Mortality and morphological abnormalities were also recorded to determine the safe concentration range.

### 4.4. Establishment of a Model of Osteoporosis of Zebrafish Induced by Glucocorticoids

Tg(ola.*sp7*:nlsGFP) transgenic zebrafish larvae were used to establish a GIOP model. From 3 dpf, larvae in the model group were exposed to embryo medium containing 25 μMPred with 0.1% DMSO, while the control group received an equal volume of vehicle. Treatment was continued until 7 dpf, with the medium replaced every 24 h.

At 7 dpf, craniofacial skeletal regions of zebrafish larvae were imaged under consistent exposure conditions using a Leica M165C stereomicroscope (Wetzlar, Germany), focusing on the cleithrum (CB), branchial arches (BR), and operculum (OP). Images were quantitatively analyzed using ImageJ software (v1.54p). All images were converted to grayscale and processed under identical threshold conditions to measure fluorescence area and mean fluorescence intensity, with background subtraction applied. Data were subsequently analyzed using GraphPad Prism (v9.4.1).

### 4.5. IBA’s Intervention in Osteoporosis Model

Based on the established model, zebrafish were divided into control, model, positive control, and IBA treatment groups (*n* = 30 per group). The model group was exposed to 25 μM Pred from 3 dpf. The positive control group received etidronate disodium (10 μg/mL) under model conditions. The IBA treatment groups were exposed to 1, 2.5, and 5 μM IBA under the same conditions. All treatments were administered from 3 to 7 dpf, with medium replacement every 24 h. At 7 dpf, skeletal fluorescence imaging and quantitative analysis were performed as described in Section 2.4.

### 4.6. LY294002 Intervention Experiment

To investigate the involvement of the PI3K/Akt signaling pathway in the protective effects of IBA, LY294002 was introduced into the osteoporosis model. The experiment included five groups: control, model, LY294002 group (model + 10 μM LY294002), IBA group (model + 5 μM IBA), and combined treatment group (model + LY294002 + IBA). Treatment conditions and duration were consistent with those described above. Skeletal fluorescence imaging and quantitative analysis were performed at 7 dpf to evaluate differences in fluorescence area and intensity among groups (*n* = 30 per group).

### 4.7. Network Pharmacological Analysis

#### 4.7.1. Potential Target Screening

The chemical structure and physicochemical information of IBA (CAS No. 31524-62-6) were obtained from the PubChem (https://pubchem.ncbi.nlm.nih.gov/, accessed on 3 June 2025) database. The standardized structure file was imported into the SwissTargetPrediction (http://www.swisstargetprediction.ch/, accessed on 13 June 2025) and TCMSP (https://www.tcmsp-e.com/load_intro.php, accessed on 13 June 2025) databases for potential target prediction. In SwissTargetPrediction, the species was restricted to Homo sapiens, and targets with probability > 0 were retained. The TCMSP database was used to supplement reported and predicted targets. After merging and removing duplicate entries, the targets were standardized using the UniProt database (https://www.uniprot.org/, accessed on 13 June 2025), with species restricted to Homo sapiens and converted to official gene symbols. Unannotated or non-human entries were excluded.

#### 4.7.2. Overlap Target Screening, PPI Network Construction, GO/KEGG Enrichment Analysis, and Target–Pathway Network Visualization

To acquire the common drug–disease targets, the overlapping targets between IBA and OP were first determined. A Venn diagram produced with the Venny online tool (v2.1.0, https://bioinfogp.cnb.csic.es/tools/venny/index.html, accessed on 9 June 2025) was used to display these common targets.

These shared targets were then loaded into the STRING database (https://string-db.org/, accessed on 10 June 2025) under the species parameter Homo sapiens, allowing the construction of a protein–protein interaction (PPI) network. The resulting PPI network was imported into Cytoscape software (v3.10.1) for topological analysis. Degree value was used as the primary parameter to evaluate node importance within the network. The top ten targets ranked by degree value were selected as hub targets for subsequent analyses.

For functional enrichment analyses, the above-identified common drug–disease targets were submitted to the DAVID Bioinformatics Resources (Database for Annotation, Visualization, and Integrated Discovery; https://davidbioinformatics.nih.gov/, accessed on 25 June 2025). Both Gene Ontology (GO) functional enrichment and Kyoto Encyclopedia of Genes and Genomes (KEGG) pathway enrichment were carried out with the species restricted to Homo sapiens. A significance threshold of *p* < 0.05 was adopted. Based on the *p*-values, the enriched biological processes were transformed by negative logarithmic conversion (−log *P*) and then ranked in descending order. For visualization, the ten most significant GO terms (covering BP, CC, and MF) and the twenty KEGG pathways that contained the largest number of common targets were selected. Bar plots and bubble plots were generated to present the enrichment results. Subsequently, a target–pathway network was built to pinpoint the targets that appeared most frequently across the pathways, and the network was visualized accordingly.

### 4.8. Molecular Docking

The three-dimensional structures of target proteins were obtained from the RCSB PDB database (https://www.rcsb.org/, accessed on 25 June 2025), prioritizing crystal structures with resolution ≤ 2.5 Å and co-crystallized ligands. Protein structures were processed using PyMOL (v2.5.0) to remove water molecules and non-essential ligands, followed by hydrogen addition. AutoDock Tools (v1.5.7) was used to prepare PDBQT files. The structure of IBA was obtained from PubChem (https://pubchem.ncbi.nlm.nih.gov/, accessed on 28 June 2025) in SDF format and converted to PDBQT format after geometry optimization and energy minimization using ChemBio3D (Ultra 14.0).

Molecular docking was performed using AutoDock Vina (v1.1.2). Both blind docking and site-specific docking based on known active pockets were conducted. Grid box parameters were defined according to ligand-binding sites, and exhaustiveness was set to 8. Each docking process was repeated 20 times to improve reliability. The optimal binding conformations were selected based on binding energy (kcal/mol), conformational stability, and structural rationality. Docking scores generated by the Vina scoring function were used to evaluate the predicted binding affinity between IBA and target proteins.

### 4.9. MD Simulation

MD simulations were performed using the GROMACS 2022 package for 100 ns. The Amber14SB force field was applied to proteins, and the GAFF2 force field was used for ligands. RESP charges were calculated using the AmberTools22 package. The complex was placed in a cubic TIP3P water box with a minimum distance of 1.0 nm from the box edge, and 0.15 M NaCl was added to neutralize the system.

Energy minimization was performed using the steepest descent algorithm until the maximum force was <1000 kJ/(mol·nm). This was followed by 100 ps NVT equilibration at 310 K using a V-rescale thermostat, and 100 ps NPT equilibration at 1 bar. The production simulation was then conducted for 100 ns with a time step of 2 fs. Long-range electrostatic interactions were calculated using the PME method, and the van der Waals cutoff was set to 1.0 nm. All hydrogen bonds were constrained using the LINCS algorithm. Trajectory data were recorded every 10 ps. RMSD, RMSF, Rg, SASA, and hydrogen bonds were calculated to evaluate system stability.

### 4.10. RT-qPCR Verification

Samples were collected at 7dpf under the same experimental conditions as described above. For each group, 30 larvae were pooled as one biological replicate, and three independent biological replicates were performed. Each qPCR reaction was conducted in triplicate as technical replicates.

Total RNA was extracted using TRIzol, and RNA concentration and purity were determined. Reverse transcription was performed using 2 μg RNA. Quantitative real-time PCR was conducted using a CFX96 system with SYBR Green chemistry. The reaction volume was 20 μL, including 10 μL 2× premix, 1 μL primer mixture, and 9 μL diluted cDNA. Each sample was analyzed in technical triplicate.

Target genes included *esr1*, *ccnd1*, *alpl*, *opn*, *sp7*, *runx2*, and *bglap*. *β-actin* was used as the internal reference gene. The primer sequences used in the experiments was shown in Table 3. Relative expression levels were calculated using the 2^−ΔΔCt^ method. Statistical analysis was performed using one-way ANOVA followed by Tukey’s post hoc test. A value of *p* < 0.05 was considered statistically significant.

## 5. Conclusions

This study systematically confirmed that IBA exerts a significant protective effect against glucocorticoid-induced osteoporosis in a zebrafish model. By upregulating the mRNA expression of a comprehensive panel of key osteogenic genes, including *alpl*, *spp1*, *sp7*, *runx2* and *bglag*, IBA effectively reverses the bone mineralization damage caused by Pred. Mechanistically, our findings reveal that IBA functions as a ligand-mimetic that activates the ESR1-PI3K/Akt signaling axis, thereby driving the programs for osteoblast differentiation and subsequent bone formation. The indispensability of this pathway was further validated through PI3K-inhibitor (LY294002) rescue experiments, which demonstrated that blocking this signaling hub abolishes IBA’s osteoprotective efficacy. In summary, this study provides preclinical evidence and mechanistic insights supporting IBA as a promising natural flavonoid candidate for the development of multi-target therapeutic strategies against secondary osteoporosis and related metabolic bone disorders, with potential translational value in endocrine-associated skeletal diseases.

## Figures and Tables

**Table 1:** Annotation of KEGG pathways.

Term ID	Description	Count	*p* Value	Genes
hsa05206	MicroRNAs in cancer	12	0.00012812372183936	*ABCC1*, *CCND1*, *MMP16*, *PLAU*, *ERBB2*, *ABL1*, *CYP1B1*, *MDM4*, *RAF1*, *PTGS2*, *MET*, *MTOR*
hsa05010	Alzheimer disease	12	0.000883117386169321	*BACE1*, *GSK3B*, *APP*, *ADAM17*, *NOS2*, *NOX4*, *BRAF*, *CALM3*, *CALML3*, *RAF1*, *PTGS2*, *MTOR*
hsa04151	PI3K-Akt signaling pathway	11	0.001761574158878574	*RET*, *GSK3B*, *HSP90AA1*, *RXRA*, *SYK*, *CCND1*, *ERBB2*, *CDK2*, *RAF1*, *MET*, *MTOR*
hsa04080	Neuroactive ligand-receptor interaction	11	0.0020706265242342774	*OPRD1*, *PRSS1*, *GLRB*, *GCGR*, *ADORA3*, *ADORA1*, *AGTR1*, *TRPV1*, *ADRB2*, *OPRM1*, *ADRA2A*
hsa05022	Pathways of neurodegeneration—multiple diseases	11	0.013183235695435625	*GSK3B*, *APP*, *NOS2*, *NOX4*, *BRAF*, *CALM3*, *CALML3*, *RAF1*, *MAPK14*, *PTGS2*, *MTOR*
hsa05167	Kaposi sarcoma-associated herpesvirus infection	10	0.000075949588239302	*GSK3B*, *SYK*, *CCND1*, *SRC*, *CALM3*, *CALML3*, *RAF1*, *MAPK14*, *PTGS2*, *MTOR*
hsa05417	Lipid and atherosclerosis	10	0.000159670041632707	*GSK3B*, *HSP90AA1*, *RXRA*, *SRC*, *MMP3*, *CALM3*, *CALML3*, *PPARG*, *MAPK14*, *SOD2*
hsa05163	Human cytomegalovirus infection	10	0.000224420137771673	*GSK3B*, *CCND1*, *SRC*, *CALM3*, *CALML3*, *RAF1*, *MAPK14*, *PTGS2*, *B2M*, *MTOR*
hsa05208	Chemical carcinogenesis—reactive oxygen species	10	0.00023195140446227	*PTPN1*, *SRC*, *ABL1*, *CYP1B1*, *NOX4*, *BRAF*, *RAF1*, *MAPK14*, *SOD2*, *MET*
hsa04915	Estrogen signaling pathway	9	0.0000398860428061955	*HSP90AA1*, *SRC*, *CALM3*, *CALML3*, *OPRM1*, *RAF1*, *ESR1*, *CTSD*, *ESR2*
hsa05226	Gastric cancer	9	0.0000686400105856275	*GSK3B*, *RXRA*, *CCND1*, *ERBB2*, *CDK2*, *BRAF*, *RAF1*, *MET*, *MTOR*
hsa04022	cGMP-PKG signaling pathway	9	0.000139685482828282	*OPRD1*, *ADORA3*, *ADORA1*, *AGTR1*, *CALM3*, *CALML3*, *ADRB2*, *RAF1*, *ADRA2A*
hsa04081	Hormone signaling	9	0.000901817324218368	*OPRD1*, *SRC*, *GCGR*, *AGTR1*, *ADRB2*, *OPRM1*, *ESR1*, *ESR2*, *ADRA2A*
hsa04371	Apelin signaling pathway	8	0.000298020786930806	*CCND1*, *NOS2*, *SERPINE1*, *AGTR1*, *CALM3*, *CALML3*, *RAF1*, *MTOR*
hsa05224	Breast cancer	8	0.000417973799301794	*GSK3B*, *CCND1*, *ERBB2*, *BRAF*, *RAF1*, *ESR1*, *ESR2*, *MTOR*
hsa04218	Cellular senescence	8	0.000596197027254038	*CCND1*, *CDK2*, *SERPINE1*, *CALM3*, *CALML3*, *RAF1*, *MAPK14*, *MTOR*
hsa05152	Tuberculosis	8	0.0014212822988480985	*SYK*, *NOS2*, *SRC*, *CALM3*, *CALML3*, *RAF1*, *MAPK14*, *CTSD*
hsa04020	Calcium signaling pathway	8	0.008922515546162545	*RET*, *NOS2*, *ERBB2*, *AGTR1*, *CALM3*, *CALML3*, *ADRB2*, *MET*
hsa04917	Prolactin signaling pathway	7	0.000046096183355707	*GSK3B*, *CCND1*, *SRC*, *RAF1*, *MAPK14*, *ESR1*, *ESR2*
hsa05223	Non-small cell lung cancer	7	0.0000539921503905596	*RET*, *RXRA*, *CCND1*, *ERBB2*, *BRAF*, *RAF1*, *MET*

**Table 2:** Molecular docking bonding energy and binding site data.

Target Name	Binding Energy (kcal·mol^−1^)	Binding-Site Residues and Hydrogen Bond Lengths (Å)
AKT1	−9.1	THR211 (2.93, 3.9); ILE290 (2.21, 2.86)
CCND1	−9.3	VAL96 (2.73, 3.66); VAL96 (2.28, 2.87); ASP158 (2.51, 3.41)
ESR1	−9.5	GLU75 (3.81, 4.55); GLU2058 (3.75, 2.87)
GSK3B	−9.5	VAL135 (2.26, 3.19); ASP200 (2.08, 2.91)
mTOR	−10.4	GLU85 (2.91, 3.85); GLU2032 (3.2, 3.99); SER2035 (3.2, 3.73)
PIK3CA	−8.1	GLU353 (2.79, 3.22); ARG394 (2.79, 3.64); HIS524 (2.52, 3.27) LEU525 (2.63, 2.94)

**Table 3:** Sequence of primers for the RT-qPCR analysis.

Gene	Forward Sequence (5′→3′)	Reverse Sequence (5′→3′)
*esr1*	CCAGCCTGTAATGGGACTCA	TCTCTCTCAGGAATCGGGCT
*ccnd1*	ACTTCCTTGCCAAACTGCCT	TGAAGTTGACGTCTGTCGCA
*alpl*	GGCAAATCAGTGGGAATCGTC	CATTGGGCATGTCTGCATCAG
*opn*	AAACTGCACTACCCCTGAGC	CAGCATTGTACGTCGGTGGA
*sp7*	CCAGACCTCCAGTGTTTCCC	GCTTGTAAGGCAATCCGCAG
*runx2*	ACTCCTAACCTAAAAGGCGTCA	GCTGACATGGGGTCACAGAA
*bglap*	ACCTGACTCCATTTCAGCTCG	CGATGATTCCAGACGTGTCCA
*β-actin*	GCCAACAGAGAGAAGATGACACAG	CAGGAAGGAAGGCTGGAAGAG

## Data Availability

The datasets used in this study are available from the corresponding authors upon reasonable request.

## References

[1] Buckley L., Humphrey M.B. (2018). Glucocorticoid-induced osteoporosis. N. Engl. J. Med..

[2] Compston J.E., McClung M.R., Leslie W.D. (2019). Osteoporosis. Lancet.

[3] Yunusoğlu O., Koyuncu E. (2025). Alpha-lipoic acid in pharmaceutical development: A comprehensive review of its therapeutic potential and molecular mechanisms. Prospect. Pharm. Sci..

[4] Mohammed F.S., Sevindik M., Uysal İ., Sabik A.E. (2023). Quercetin: Derivatives, biosynthesis, biological activity, pharmacological and therapeutic effects. Prospect. Pharm. Sci..

[5] Dharani B., Suba A. (2026). A novel targeted formulation for osteoarthritis: Exploring synergistic benefits of Cissus quadrangularis, Boswellia serrata, propolis and palmitoylethanolamide. Prospect. Pharm. Sci..

[6] Xin C., Zhang G., Shen Z., Han W., Fan R., Ren J., Zhang J., Hao Y., Xin J. (2025). Pharmacological mechanism of natural products to treat osteoporosis: A focus on the autophagy. Front. Pharmacol..

[7] Guo A.J., Choi R.C., Zheng K.Y., Chen V.P., Dong T.T., Wang Z.-T., Vollmer G., Lau D.T., Tsim K.W.-k. (2012). Kaempferol as a flavonoid induces osteoblastic differentiation via estrogen receptor signaling. Chin. Med..

[8] Li T., Du Y., Yao H., Zhao B., Wang Z., Chen R., Ji Y., Du M. (2024). Isobavachin attenuates osteoclastogenesis and periodontitis-induced bone loss by inhibiting cellular iron accumulation and mitochondrial biogenesis. Biochem. Pharmacol..

[9] Noor F., Tahir Ul Qamar M., Ashfaq U.A., Albutti A., Alwashmi A.S.S., Aljasir M.A. (2022). Network Pharmacology Approach for Medicinal Plants: Review and Assessment. Pharmaceuticals.

[10] Chao P., Zhang X., Zhang L., Yang A., Wang Y., Chen X. (2024). Integration of molecular docking and molecular dynamics simulations with subtractive proteomics approach to identify the novel drug targets and their inhibitors in *Streptococcus gallolyticus*. Sci. Rep..

[11] Tonelli F., Bek J.W., Besio R., De Clercq A., Leoni L., Salmon P., Coucke P.J., Willaert A., Forlino A. (2020). Zebrafish: A resourceful vertebrate model to investigate skeletal disorders. Front. Endocrinol..

[12] DeLaurier A., Eames B.F., Blanco-Sánchez B., Peng G., He X., Swartz M.E., Ullmann B., Westerfield M., Kimmel C.B. (2010). Zebrafish sp7: EGFP: A transgenic for studying otic vesicle formation, skeletogenesis, and bone regeneration. Genesis.

[13] Liu Q., Li M., Wang S., Xiao Z., Xiong Y., Wang G. (2020). Recent advances of osterix transcription factor in osteoblast differentiation and bone formation. Front. Cell Dev. Biol..

[14] Du J., Wang Y., Wu C., Zhang X., Zhang X., Xu X. (2024). Targeting bone homeostasis regulation: Potential of traditional Chinese medicine flavonoids in the treatment of osteoporosis. Front. Pharmacol..

[15] Koromila T., Baniwal S.K., Song Y.S., Martin A., Xiong J., Frenkel B. (2014). Glucocorticoids antagonize RUNX2 during osteoblast differentiation in cultures of ST2 pluripotent mesenchymal cells. J. Cell. Biochem..

[16] Feng X. (2009). Chemical and biochemical basis of cell-bone matrix interaction in health and disease. Curr. Chem. Biol..

[17] Long F. (2012). Building strong bones: Molecular regulation of the osteoblast lineage. Nat. Rev. Mol. Cell Biol..

[18] Mckee M.D., Nanci A. (1996). Osteopontin: An interfacial extracellular matrix protein in mineralized tissues. Connect. Tissue Res..

[19] Almeida M., Iyer S., Martin-Millan M., Bartell S.M., Han L., Ambrogini E., Onal M., Xiong J., Weinstein R.S., Jilka R.L. (2013). Estrogen receptor-α signaling in osteoblast progenitors stimulates cortical bone accrual. J. Clin. Investig..

[20] So M.C., Hwang H.P., Lee C.H., Youn H.J., Jung S.H., Kim J.C. (2006). Up-regulation of Pi3k/Akt Signaling by 17β-estradiol through Activation Of Estrogen Receptor-α in Breast Cancer Cells. J. Breast Cancer.

[21] Saczko J., Michel O., Chwiłkowska A., Sawicka E., Mączyńska J., Kulbacka J. (2017). Estrogen receptors in cell membranes: Regulation and signaling. Adv. Anat. Embryol. Cell Biol..

[22] Dong J., Xu X., Zhang Q., Yuan Z., Tan B. (2020). The PI3K/AKT pathway promotes fracture healing through its crosstalk with Wnt/β-catenin. Exp. Cell Res..

[23] Liu C., Zhang J., Ye Z., Luo J., Peng B., Wang Z. (2025). Research on the role and mechanism of the PI3K/Akt/mTOR signalling pathway in osteoporosis. Front. Endocrinol..

[24] Shaikh A., Wesner A.A., Abuhattab M., Kutty R.G., Premnath P. (2023). Cell cycle regulators and bone: Development and regeneration. Cell Biosci..

[25] Jin X., Sun J., Yu B., Wang Y., Sun W.J., Yang J., Huang S.H., Xie W.L. (2017). Daidzein stimulates osteogenesis facilitating proliferation, differentiation, and antiapoptosis in human osteoblast-like MG-63 cells via estrogen receptor–dependent MEK/ERK and PI3K/Akt activation. Nutr. Res..

[26] Zhao B., Chen L., Wang W., Xu W., Xu B. (2025). Anti-Osteoporosis Activity of Lycopene Through ESR1: Network Pharmacology, Molecular Docking, Imaging Technology, and Experimental Validation. Chem. Biol. Drug Des..

[27] Hu H.-Y., Zhang Z.-Z., Jiang X.-Y., Duan T.-H., Feng W., Wang X.-G. (2023). Hesperidin anti-osteoporosis by regulating estrogen signaling pathways. Molecules.

[28] Chung Y.C., Song S.J., Lee A., Jang C.H., Kim C.-S., Hwang Y.-H. (2024). Isobavachin, a main bioavailable compound in Psoralea corylifolia, alleviates lipopolysaccharide-induced inflammatory responses in macrophages and zebrafish by suppressing the MAPK and NF-κB signaling pathways. J. Ethnopharmacol..

[29] Wang Y., Li B., Zhang W., Liu Y., Xue P., Ma J., Li Y. (2013). Impaired PI3 K Akt expression in liver and skeletal muscle of ovariectomized rats. Endocrine.

[30] Ghosh R., Colon-Negron K., Papa F.R. (2019). Endoplasmic reticulum stress, degeneration of pancreatic islet β-cells, and therapeutic modulation of the unfolded protein response in diabetes. Mol. Metab..

[31] Ribas V., Drew B.G., Zhou Z., Phun J., Kalajian N.Y., Soleymani T., Daraei P., Widjaja K., Wanagat J., de Aguiar Vallim T.Q. (2016). Skeletal muscle action of estrogen receptor α is critical for the maintenance of mitochondrial function and metabolic homeostasis in females. Sci. Transl. Med..

